# The impact of perceived similarity on tacit coordination: propensity for matching and aversion to decoupling choices

**DOI:** 10.3389/fnbeh.2015.00202

**Published:** 2015-07-28

**Authors:** Gabriele Chierchia, Giorgio Coricelli

**Affiliations:** ^1^Department of Social Neuroscience, Max Planck Institute for Human Cognitive and Brain ScienceLeipzig, Germany; ^2^Center for Mind/Brain Science, University of TrentoRovereto, Italy; ^3^Economics Department, University of Southern CaliforniaLos Angeles, CA, USA

**Keywords:** coordination, similarity, homophily, economic games, social preferences, social cognition

## Abstract

Homophily, or “love for similar others,” has been shown to play a fundamental role in the formation of interpersonal ties and social networks. Yet no study has investigated whether perceived similarities can affect tacit coordination. We had 68 participants attempt to maximize real monetary earnings by choosing between a safe but low paying option (that could be obtained with certainty) and a potentially higher paying but “risky” one, which depended on the choice of a matched counterpart. While making their choices participants were mutually informed of whether their counterparts similarly or dissimilarly identified with three person-descriptive words as themselves. We found that similarity increased the rate of “risky” choices only when the game required counterparts to match their choices (stag hunt games). Conversely, similarity led to decreased risk rates when they were to tacitly decouple their choices (entry games). Notably, though similarity increased coordination in the matching environment, it did not did not increase it in the decoupling game. In spite of this, similarity increased (expected) payoffs across both coordination environments. This could shed light on why homophily is so successful as a social attractor. Finally, this propensity for matching and aversion to decoupling choices was not observed when participants “liked” their counterparts but were dissimilar to them. We thus conclude that the impact of similarity of coordination should not be reduced to “liking” others (i.e., social preferences) but it is also about predicting them.

## Introduction

“Any event in the history of the organism is, in a sense, unique. Consequently, recognition, learning, and judgment presuppose an ability to categorize stimuli and classify situations by similarity. As Quine (1969) puts it: “There is nothing more basic to thought and language than our sense of similarity; our sorting of things into kinds” (Tversky and Gati, 1978).

So many of our decisions are “social”: their outcomes depend on the decisions of others. This can generate “strategic uncertainty” (Van Huyck et al., 1990) and require agents to infer what others will do, while others do the same, in order to decide optimally.

Consider for instance the decision of joining a strike or a rebellion: all may know that if enough people join in the uprising, it will succeed, and everyone will benefit. However, rebelling in small numbers could be dangerous, so agents might hesitate to do so. Similarly, investing in a new technology might only worthwhile if enough others do the same, so it becomes the new standard and everyone profits. In such situations, two outcomes would do: “either all rebel/invest, or no one does,” but isolated actions are costly. Consequently, agents should attempt to *match* their choices.

Conversely, there are many situations in which choosing the same options can be disadvantageous, especially when resources cannot be shared. For instance, many markets can only provide revenue to a limited number of investors, because if too many do there will be a “price war,” and everyone loses (Camerer, 2003). Or more mundanely, driving space is limited, so a driver deciding whether to enter the freeway around rush hour might only do so if he convinces himself that not too many others will do the same, since if too many do, there will be a traffic jam^1^. In these situations, agents would prefer to *decouple* their choices, such that “either I take the free-way/enter the market and you don't, or vice versa; but we shouldn't enter together.”

In economics, the first class of situations are said to involve strategic complements, while the latter involve strategic substitutes (Bulow et al., 1985; Camerer and Fehr, 2006)^2^. In both cases however, when communication is impossible or inefficient^3^ (Morris and Shin, 2002; Heinemann et al., 2004), agents must find some “tacit agreement” on how to *coordinate* their choices.

Game theory is a standard approach to understanding interdependent decision problems (henceforth, “games”), and it is frequent practice in economics and political science to use it to provide strategic advice to investors, firms, or nations (Schelling, 1960; Gibbons, 1992). However, when it comes to a certain subset of interactions, namely “coordination problems” like the ones sketched above, game theory turns strikingly blind. This occurs because game theory fundamentally derives its predictions by applying deduction to the incentives and options of a given situation. Indeed, out of all the possible outcomes of an interaction, there is only a subset of them, called “Nash equilibria,” in which no agent has an incentive to “move further,” that is, to unilaterally deviate from his/her current choice. The fundamental problem with coordination games is that they have *multiple* Nash equilibria, and standard game theory provides no clear criteria for equilibrium selection^4^. Indeed, coordination has been said to constitute “the hardest problem of game theory” (Camerer, 2003).

Here, we investigated how coordination is affected by perceived interpersonal similarities. Indeed, homophily, or “love for similar others,” is one of the most strikingly ubiquitous predictors of interpersonal attraction and network formation in social species, as it has been observed across ages (i.e., Meltzoff, 2007; Over et al., 2013), cultures (Apicella et al., 2012) and species (i.e., Seyfarth and Cheney, 2012; Massen and Koski, 2014). Indeed, similarity along a wide variety of dimensions such as age, ethnicity, class, religion, personality and interests has been shown to shape friendship formation, partner selection and social networks in human adults (see McPherson et al., 2001 for a review)^5^. Correspondingly, a number of theoretical models have implicated similarity in the formation of friendship (Currarini et al., 2009) or the evolution of cooperation (Riolo et al., 2001); and simulations have shown how, in repeated cooperation dilemmas, agents that rely on a “perceived similarity index” can drive groups of stochastic or hostile “free-riders” to extinction, even if in minority (Fischer et al., 2013). Finally, studies have begun to emphasize the impact of similarity on coordination (Cole and Teboul, 2004; Fischer, 2009; Fu et al., 2012). Yet in spite of this mounting evidence, no study has empirically assessed whether coordination is actually affected by perceived interpersonal similarities.

Furthermore, no study has systematically compared the impact of similarity on the two opposite “declinations” of coordination illustrated above, namely matching (strategic compliments) and decoupling choices (strategic substitutes). In fact, common intuition suggests that similarity should generally decrease social uncertainty, plausibly because all else being equal, similar agents can use “their own minds” as a proxy to predict the choices of their counterpart. Indeed, this is in line with abundant experimental evidence showing that, even in the absence of similarity-related cues (and since childhood) social inferences are often contaminated by one's own thoughts and perspectives (i.e., Ross et al., 1977; Wimmer and Perner, 1983; Baron-Cohen et al., 1985; Keysar, 1994; Gilovich et al., 1998, 2000; Goldman, 2006)^6^, and that perceived interpersonal similarities accentuate the degree to which this occurs (Clement and Krueger, 2002; Ames, 2004; Epley et al., 2004; Robbins and Krueger, 2005)^7^.

The problem is that being more predictable might indeed help in environments that require agents to match their choices, but it might even be detrimental when agents are to somehow “outsmart” one another and decouple their choices. For instance, in competitive environments similar agents might find it harder to manipulate or lie to one another, thus similarity might deter them from attempting to do so. Or one could think of the paradigmatic form of interpersonal similarity, namely, monozygotic twins. In fact, twins have been shown to more frequently match their choices in cooperation dilemmas (Segal and Hershberger, 1999), however, what would happen if they were to play a game like “rock, paper, scissors”?^8^ The same similarity that helped them in the first scenario, might work against them in the latter, and Fischer (2009) nicely illustrates this problem with a mental experiment involving agents “playing” with their “mirror image.” Indeed, though it has been previously conjectured (Fischer, 2009; Fu et al., 2012; Krueger et al., 2012) that similarity could affect coordination in opposite ways, this has been never empirically demonstrated.

However, and importantly, similarity doesn't only alter social inferences. In fact, traditionally, it is mainly held to mediate interpersonal attraction (Byrne, 1971; McPherson et al., 2001; Montoya et al., 2008 for a review), thus moderating social attitudes and affect. In this sense, attraction for similar others could hinge on very basic and relatively “non-inferential” mechanisms (Zajonc, 1980; Mitchell et al., 2006) such as the “mere exposure” effect (see Zajonc, 2001 for review), which consists in the observation that simple repeated exposure to previously neutral stimuli increases their perceived attractiveness (Monahan et al., 2000)^9^.

Indeed, many effects of similarity on interactions could be potentially be explained by “social preference” theories (i.e., Fehr and Fischbacher, 2003; or Camerer, 2003 for a review), which are fundamentally different from the ones described above. In fact, proponents of a “similarity approach” (Ames, 2004; Fischer, 2009; Krueger et al., 2012; Fischer et al., 2013) usually refer to its impact on inferences and uncertainty, while social preferences, if taken rigorously, leave inferences untouched, and explain cooperation in terms, for instance, of “altruistic” motives (i.e., Van Lange, 1999). The difference is that in the latter case, subjects might be willing to incur more costs to benefit those they like rather than dislike (i.e., Jones and Rachlin, 2006), thus choosing the options that most benefit similar rather than dissimilar others.

Intriguingly, alternative evolutionary approaches to homophily seem to parallel this dichotomy (albeit naturally at a much more distal level). For instance, in line with a “preference” approach to similarity, a commonly quoted evolutionary basis for homophily is kinship selection (Hamilton, 1963), the notion that agents may have an incentive to benefit others proportionally to their genetic relatedness. Indeed, by helping relatives, agents promote the survival of the portion of genes they share with them. Similarity could then be involved in distinguishing kin from non-kin. For instance phenotypic matching (Porter, 1987)—that is, the implicit evaluation of relatedness based on phenotypic similarity—has been observed in ground squirrels (Holmes and Sherman, 1982), baboons (Alberts, 1999), rhesus monkeys, and a number of other species. In line with this, DeBruine (2002) showed that economic trust was increased when human agents played with a fictive player whose face had been morphed to physically resemble their own. Intriguingly, even genotypic homophily has been reported in humans (Fowler et al., 2011; Christakis and Fowler, 2014), such that friends are more likely to have similar genes, plausibly as a consequence of their seeking others with similar phenotypes. Importantly, kin-selection based explanations of homophily imply that individuals needn't directly benefit from similarity, if anything, their genes do.

On the other hand, different evolutionary approaches related to homophily seem to stress its predictive or strategic component, rather than its motivational one (Fu et al., 2012; Fischer et al., 2013). For instance, Vallortigara and Rogers (2005), were concerned by the fact that selection pressures on the individual cannot explain the fact that, at the population level, the great majority of vertebrates exhibit functional lateralization in proportions that are different from 1/2. For instance, there is no clear fitness advantage of being right or left-handed, yet humans are most frequently right-handed. The authors show how this might emerge as an evolutionary stable strategy when asymmetric organisms must coordinate their behavior with other asymmetric organisms of the same species (Ghirlanda and Vallortigara, 2004). Intriguingly, there is some evidence that species that are “less social” also exhibit less population-level lateralization (Vallortigara and Bisazza, 2002). In apparent contrast to kin-selection, homophily under this approach more clearly benefits both actors and counterparts, because it is the solution to a fundamental coordination problem (in this case, functional lateralization) stemming from the necessity to predict behavior Vallortigara and Rogers (2005).

However, similarity and liking seem deeply intertwined^10^, so we also asked what aspect of similarity may have an impact on coordinated behavior; or differently put, is similarity just one among the many mediators of social attraction, or is there some additional strategic/inferential benefit to interacting with similar others, that would be harder to obtain from social attitudes alone?

In synthesis, this study asks three questions. The main one is whether perceived similarities can have an impact on tacit coordination. The second is whether this impact is the same in coordination environments requiring players to either match or decouple their choices. The third asks what aspect of similarity might guide coordination: the fact that agents simply “like” similar others, or the fact that they are better at predicting them? To shed light on these questions, we varied similarity and liking independently and assessed their separate contributions to coordination problems involving strategic complements and substitutes.

## Methods

### The games

We used two types of (two-player) coordination games: “Stag Hunts” (SHs) and “Entry Games” (EGs), which have been extensively studied, both in theory and experimental settings (reviewed in Camerer, 2003). In our versions—adapted from Heinemann et al. (2009)—we attempted to keep the superficial aspects of the two games as similar as possible, so that any behavioral difference would be due to their structural (incentive-related) differences. The two games were played in randomized order, and were as follows: in both, two agents had to choose between the same two options: (1) a potentially high paying but uncertain payoff (“UP”), always worth either $/€15.00 or 0; and (2) a lower paying but safe payoff (“SP”), worth a given $ amount (with SP ≤ 15.00). Both games capture a frequent situation, namely, that low gains can be obtained safely in isolation, while high paying outcomes involve coordination and uncertainty. Indeed, in both games, if the SP was chosen, it was obtained for sure, regardless the choice of one's counterpart. On the other hand, the outcome of choosing the UP depended on the choice of one's counterpart, and on the game: in SHs, $15.00 were obtained, by both players, if, and only if, both chose the uncertain option; thus, if only one chose the UP, he or she obtained 0. In EGs, on the other hand, the high gain could *only* be obtained in isolation, thus if both players chose the potentially high paying UP, both obtained 0. It follows that in SHs, the incentives induce players to attempt and match their choices (either both “risk” and choose the UP, or neither should, but mismatching is costly), while in EGs, players should try to decouple their choices (such that either one player “risks” and the other doesn't, or vice versa, but players should not risk together). Then, by progressively increasing the value of the SP and having participants choose at each (randomized) step we obtained a measure of their uncertainty in the two games; that is, of their willingness to choose the uncertain option, over the lower but certain one. Importantly, since initial coordination patterns usually determine the outcome of their repeated versions (Heinemann et al., 2004), and since we were here interested in the way social distance biases choices rather than how it may bias learning, no feedback on the outcomes of decisions was provided until the end of the experiment. Notably, this set up enabled to compare two very different games by visually altering only a minor detail. Indeed, both games presented (a list of) the SP magnitudes on one side of the screen and the fixed high payoff ($15.00) on the other. The games were thus only differentiated by what was written under the high payoff. The SH condition read, “$15.00 only if 2,” and the EG read, “$15.00 if at most 1” (see the instructions in the Supplementary Material for snapshots).

### Similarity induction

**Figure 1:**
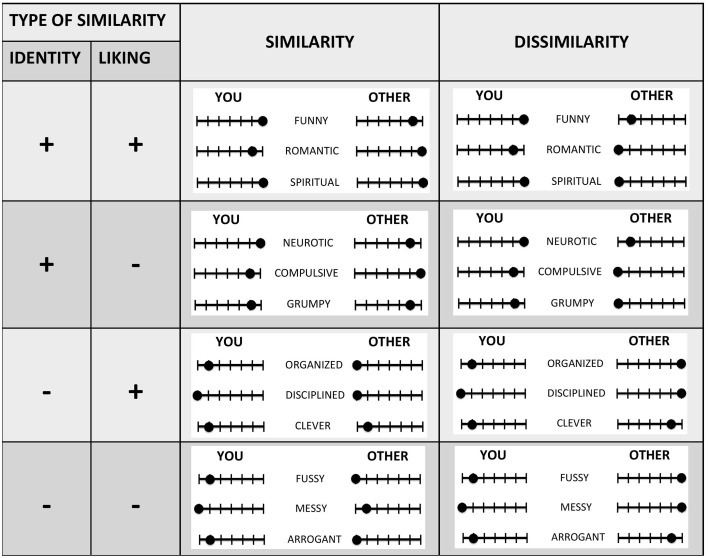
**Experimental design**. Before taking part in the interactions of interest, participants provided both “liking” and “identity” ratings of a set of 100 personality traits. Subsequently, they interacted with counterparts that were either similar or dissimilar to them with respect to a selected subset of the traits. While playing the games, matched counterparts were told that they mutually viewed rating bars indicating whether they identified or did not identify with the traits. “Liking+” and “Liking-” indicate that participants liked or disliked the selected traits.

Similarity was manipulated by making participants play with counterparts that were either similar or dissimilar to them. The similarity between players and counterparts was based on the match (similarity condition) or mismatch (dissimilarity condition) of identification ratings (on a scale from 1 to 7) with a group of (three) adjectives describing personality traits. These groups of adjectives were obtained as follows: before participants knew about the games, they rated 100 adjectives describing personality traits. They did so twice: once, indicating how much they identified with a given trait (“ID”), and the second, how much they liked the same trait (“Like”) (in counterbalanced order). As soon as they finished, an algorithm (see Table 2 in the Supplementary Material for details) went through the identity and liking ratings of each of the 100 words and retrieved four groups of words (three words per group) for each participant: (1) the first group was composed of words that a given participant both strongly identified with *and* strongly liked (i.e., maximizing both liking and identity ratings) (“ID+_Like+”); (2) the second group consisted of words that were identified with but disliked (“ID+ Like−”); (3) the third group, of words that were liked but not identified with (“ID- Like+”); while (4) the fourth and last group was composed of words that were not identified with and were disliked (“ID- Like−”) (see Figure 1). Participants then were told that they would have interacted with several anonymous counterparts and that matched counterparts would have been mutually informed of how they identified with a same group of person-descriptive words. This was made known to participants by the use of rating bars (see Figure 1 below for the design; or Figure 4 in the instructions for a screenshot of the actual task—Supplementary Material).

Ideally, we would have preferred to not deceive participants, though our design made this difficult. The main problem was that there were relatively few words, for each participant, that satisfied the requirements of our design (i.e., that maximized identity and liking ratings etc.) and these rarely matched between participants. For instance, over the 100 person-descriptive words that participants rated, there were only few of them that each participant maximally (minimally) identified with and maximally (minimally) liked (on average, 4.3 for ID+Like+ condition—s.e. 1.7 -, and 5 for the ID-Like- condition). Furthermore, there were even less optimal words for the “incongruent” word conditions (on average, 1.8 and 1.6 for the ID+Like- and ID-Like+ conditions, respectively). Within the ~15–20 participant sessions we ran, it was thus difficult to find two players that actually rated the *same* words in similar ways. Perhaps this could have been achieved with a much larger sample size, which was unavailable to us. Alternatively, we could have chosen sub-optimal words (i.e., for the ID+Like+ condition, words that participants only weakly identified with but that were actually shared by multiple participants), though we were concerned that this would have reduced any effect of similarity. In sight of these tradeoffs, we resorted to generating the identity ratings of artificial counterparts, as this allowed us to probe for effects of similarity while effectively controlling for identity and liking. Finally, in order to minimize deception, before taking part in the task of interest, participants also took part in the same two games without knowing anything about their counterparts, and were paid for one of these randomly determined trials. Consequently, participants were in fact paid for their real choices and those of their matched counterparts, though they believed that any trial could be paid. In addition to this choice-dependent payment, participants earned a $5.00 show-up fee.

In synthesis, excluding the latter “trait-neutral” trials, this set-up yielded a 2 × 2 × 2 × 2 design, with factors: (1) game (SHs vs. EGs), (2) similarity (similar vs. dissimilar); (3) liking (liked vs. disliked traits); and (4) identity (high vs. low identification with traits). The instructions we used are available in the Supplementary Material. Within each of the resulting 16 experimental cells, SPs ranged from 1 to 15 in steps of 1, for a total of 240 decisions. Given this high number of decisions, we adopted a presentation method analogous to the strategy method (Selten, 1965), in which participants viewed all 15 SP options of a cell on a single page, rather than making each decision on a separate page. Each of the resulting 16 pages/cells (with 15 decisions for each page) was presented in randomized order.

Person-descriptive words were taken from Dumas et al. (2002), a list of 844 person related adjectives and had been rated on the basis of their likableness. Since we aimed to orthogonalize identity and liking scores as much as possible, while simultaneously inducing a sufficiently strong sense of identification and liking, we needed words that were valenced but not too clearly so, since traits with extreme (high or low) valences also had the lowest likableness variance. Thus, plausibly few people would have identified with words like “dishonest,” but some may have identified with words such as “disorganized” or “whiny.” Consequently, we sorted the traits on the basis of their likableness, and took 100 words from 2 clusters: 50 from a moderately positive cluster, and 50 from moderately negative one.

### Lottery

To control for the potential impact of inter-individual differences in (non-strategic) risk attitudes, participants took part in a “lottery” condition, which took place after the strategic games. For the lottery task, participants were endowed with $5.00 additional dollars and were then allowed to make an investment on a lottery extraction with a winning probability of 2/3. Participants could invest any amount (0 included) of their $5.00 endowment. To implement the lottery, in clear sight of all, we placed two red balls and one blue ball (of equal dimension) into a hole on the top of an opaque box, and shook it. Participants were informed that, after placing their bets, a randomly designated participant would have blindly extracted a single ball from the box. If the ball was red the experimenters would have doubled participants' investments, while if the ball was blue, the investment would have been lost. We took the amount invested by each participant as a measure of their (non-strategic)-risk attitudes.

### Participants

The experiment was carried out at the University of Southern California. In 5 sessions, 68 participants took part in the 2 coordination tasks, implemented in z-Tree (Fischbacher, 2007).

### Procedures

Participants interacted in groups, from individually shielded computer cubicles. After assigning them to a random cubicle (via a bingo chip extraction), instructions were read out loud and followed on instruction sheets. Before starting, participants also took part in a questionnaire that probed their understanding of the games. The questionnaire could only be completed by correctly responding to all of its items. This enabled us to explicitly assure participants that they, and all their potential counterparts, had understood the rules of the games. All procedures were approved by local ethical committees. Our data is available upon request.

### Statistical analysis

Data was analyzed with generalized linear mixed effects models (“GLMMs”: Bolker et al., 2009), with a “bobyqa” optimization algorithm (Powell, 2009), as implemented in the lme4 package (Baayen et al., 2008), in the R environment (Venables and Smith, 2005).

#### Analysis of choice

Since our principle dependent variable was the dichotomous choice “SP” or “UP” (i.e., “risk”) we used a GLMM with a logistic link function (as also done in Heinemann et al., 2009). Our main question of interest was whether similarity affected choices oppositely in coordination games that required participants to either match (SHs) or decouple their choices (EGs), and whether this depended on how much participants liked or identified with the personality characteristics that the similarity was based on. Correspondingly, our model included the four-way interaction between the following fixed-effect terms: game^*^similarity^*^liking^*^identity (which automatically included all lower-level interactions). Furthermore, as previous research (Nagel et al., in preparation) has shown that decreasing SPs affects the likelihood of “risking” differentially in SHs and EGs, we added an additional interaction term between these two factors as a covariate. Finally, since Heinemann et al. (2009) have shown that (non-strategic) risk attitudes (i.e., as established by lotteries with known probabilities) are related to “risk” in strategic games, we further introduced the (centered) investments participants had made in the lottery condition (“lottery”), and let this interact with the game factor. In synthesis, our model included the following fixed effect terms: sure payoff ^*^game + game^*^similarity^*^liking^*^identity + lottery^*^game. At last, our model included a random intercept term to cluster choices by participant. We report the analysis of variance table of this model in the Supplementary Table 1. Then, to inspect the significant interaction constituents (when significant), we simply re-ran the model while resetting the reference point of the factor levels of interest (i.e., as also done in Kanngiesser et al., 2010).

#### Analysis of expected payoffs

As noted above, our participants were only allegedly paid for one randomly determined trial. However, to study the potential economic impact of similarity across SPs and conditions, we computed “expected payoffs” for all trials. To do so we did the following: in any condition, had a participant “*i*” chosen the SP, the specific value of the SP was attributed to *i* (since, in both games, if one chose the SP, this was obtained no matter what one's counterpart chose). If however, on a different trial, *i* had chosen the UP (i.e., to “risk”), then *i*'s payoff was determined in expected value (“EV”), given the posterior probability of being matched to someone else that also chose the UP in the same game^11^. For instance, suppose that in a given trial participant *i* chose to risk and 70% of the other players ended up doing so as well. Then, had the trial been a SH*, i*'s expected payoff, was computed as 0.7^*^15.00 [i.e., EV_*i*_ = 0.7^*^15+0^*^(1 - 0.7)], while had the trial been an EG, it was simply computed as (1 - 0.7)^*^15.00. In this way, we were able to compute expected payoffs in all trials and explore how they varied as a function of similarity.

#### Analysis of coordination rates

We define “successful coordination” as the probability of “matching choices” (on either of the two options) in the matching environment, and of decoupling choices in the decoupling one. Notably, this notion of coordination ability needn't have a 1-to-1 relation with expected payoffs. For instance, if participants had always chosen the SP in the matching environment, they would have achieved a maximum coordination rate (according to the definition above). Moreover, they would have obtained the same (maximum) coordination rate by always choosing the “risky” option. However, they would have earned much more in the second case, coordination rates being equal. Consequently, in addition to expected payoffs, we aimed to assess how similarity affected the probability of successful coordination.

To compute the probability of successful coordination we did the following: if a given participant “*i”* had chosen the “risky” option in the matching environment, then his/her probability of successful coordination it coincided with the (posterior) likelihood of being matched to someone who had *also* chosen the risky option in the corresponding condition (and for the same SP-value)^12^. Conversely, in the decoupling environment, if a subject had chosen the “risky” option, likelihood of successful coordination was computed as the mean of potential counterparts who had *not* chosen the “risky” option. In a specular fashion, if a participant had chosen the “safe” option in the matching game, it coincided with the average amount of potential counterparts who had also chosen the safe option in the corresponding condition; while, if one chose the safe option in the decoupling environment it coincided with the mean “risk rates” of one's counterparts. In this manner we computed the probability of “successful coordination” for each choice participants made (given the actual choices of all the others), and we investigated how this was affected by similarity.

## Results

### Similarity manipulation validation

**Figure 2:**
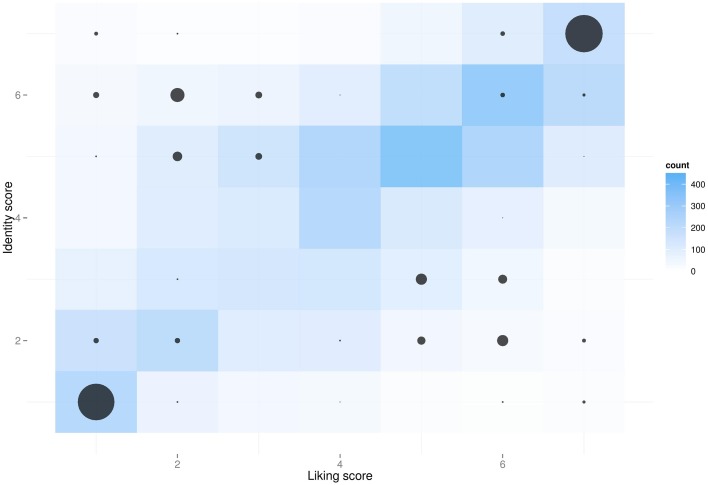
**Identity and liking ratings of 100 personality traits by 68 participants (on a 1–7 Likert scale)**. Darker blue indicates more frequent observations, which can be clearly seen to fall on the diagonal, indicating that subjects usually identify with the traits they like, and vice versa. Of the 100 traits each subject rated, an algorithm selected four subject-specific triplets of adjectives that best fit into the four corners of this “identification-by-liking” space. Black circle sizes are proportional to the number of adjectives that were selected by the algorithm. During the subsequent games, subjects were matched with others that either identified or didn't identify with the triplet of traits selected by the algorithm.

As was to be expected, we observed a positive correlation between identity and liking ratings of the 100 adjectives (*r* = 0.57, *p* < 0.001) (Figure 2, blue), suggesting that subjects usually like the traits they identify with or dislike the traits they don't identify with. In spite of this, all four groups of adjectives retrieved by our algorithm could be differentiated (Figure 2, black circles): averaged identity ratings within triplets were significantly higher for the ID+ group than the ID- group (*p* < 0.001), and liking ratings were higher in the Like+ group than the Like- group (*p* < 0.001). For the trait triplets meant to have coherent identification and liking scores (namely, ID+Like+ and ID-Like-), ratings between the two dimensions (identity and liking) were not differentiable (both *p*_*s*_ > 0.08)^13^ While for “incoherent” triplets, in which liking and identity were pitted one against the other, the two scores strongly dissociated in the anticipated directions (both *p*_*s*_ < 0.001)^14^. However, given the aforementioned correlation, liking and identity scores also slightly changed when passing from the coherent clusters (ID+Like+ and ID-Like-) to the incoherent ones (ID-Like+ and ID+Like-). For instance, liked adjectives that subjects didn't identify with (ID-Like+) were certainly liked more than disliked adjectives from either of the two “low-liking” clusters (both *p*_*s*_ < 0.001)^15^, however they weren't liked as much as those that subjects also identified with^16^. To partially alleviate this residual multicollinearity of liking and identity, we checked that the results obtained in our statistical model held when using the centered identity and liking scores, rather than the corresponding (i.e., “high vs. low”) factors.

### Behavior in games

**Figure 3:**
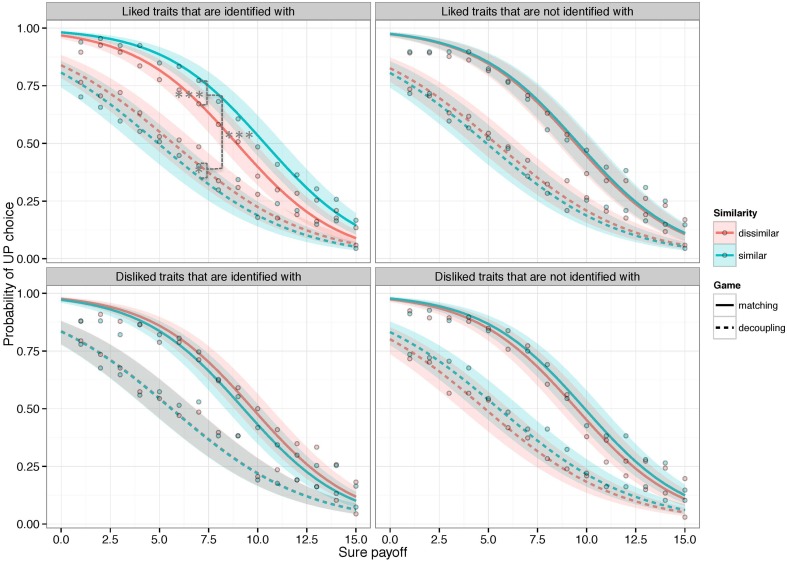
**The impact of similarity on coordination games requiring players to either match their choices (“strategic complements” – “Stag hunt” games) or decouple them (“strategic substitutes” – “Entry” games) without communicating**. Curves represent estimated probabilities of choosing a potentially higher paying, but uncertain payoff (y-axis) (“UP”), given increasing values of a safe alternative (x-axis), when interacting with either similar or dissimilar (anonymous) counterparts. Similarity was based solely on the fact that counterparts similarly identified (or did not identify) with three personality-descriptive words. Estimates were obtained with a generalized logistic mixed model. Error bands represent 95% confidence bands for the fixed effects. Points are the observed percentages of UP choices. Stars indicate significant effects of similarity on the estimated probability of making a risky choice ^*^p < 0.05, ^***^p < 0.001.

A logistic generalized mixed effects model was fit to the data and no observations were excluded from the analysis. The model revealed a significant 4-way interaction between the factors game, similarity, identity, and liking (X^2^ = 5.046, *p* < 0.05) (see Table 1 in Supplementary Material). This suggests that similarity had a differential impact on choices depending on its characteristics and on whether choices had to be matched or decoupled. The direction of the interaction constituents was as anticipated: when traits were both liked and identified (ID+Like+) similarity significantly increased the probability of “risky” choices in SHs (*p* < 0.001)^17^ but decreased it in EGs (*p* < 0.05)^18^ (Figure 3, top left panel). Notably, neither of these effects was observed when similarity was based on traits that were liked but not identified with (i.e., “we're both *not* organized” vs. “I'm not organized, you are”) (ID-Like+) (*p* = 0.22)^19^, or when similarity was based on traits that were identified with but disliked (i.e., “we're both neurotic” vs. “I'm neurotic, you are not”) (ID+Like-) (*p* = 0.91)^20^ (see Figure 3, top right and bottom left panels).

### Expected payoff

Importantly, similarity not only affected choices, but it affected expected payoffs as well. Indeed, especially when personality traits were liked and identified with (liking^*^similarity^*^identity interaction: *p* < 0.05), we observed a positive effect of similarity on payoffs, in both the SHs ($0.50, s.e. = 0.14, *p* < 0.001) and the EGs ($0.14, s.e. = 0.05, *p* < 0.01).

### Probability of successful coordination

Focusing on the condition in which we observed a significant effect of similarity on choice (the “ID+Like+” condition)^21^, we found that, in the matching environment participants in the similarity condition were indeed more likely to match their choices than in the dissimilarity condition (*p* < 0.001), especially when choosing the “risky” option. However, similarity did not increase coordination in the decoupling environment, that is, similar counterparts were *not* “better” at decoupling their choices than dissimilar ones (*p* = 0.23). More specifically, we found that this was due to the fact that, restricting the comparison of “coordination rates” to “safe choices” only, participants in the similarity condition were nearly worse at decoupling their choices than those in the dissimilarity condition (*p* = 0.07), that is, they “should have” entered more frequently. On the “flip side” of this, when restricting the same comparison to “risky choices,” participants in the similarity condition were more likely to successfully decouple their choices (*p* < 0.01).

### Covariates: game, sure payoff, and risk attitudes

As expected, our model estimated that the likelihood of making a “risky” decision was roughly 23% lower when choices had to be decoupled (EGs) rather than when they had to be matched (SHs) (*p* < 0.001), suggesting a relative propensity for matching choices, rather than decoupling them (regardless of similarity). In spite of this, uncertainty in SHs was far from absent. Indeed, while participants readily chose the uncertain option when the alternative safe payoffs were low, they gradually ceased to do so as safe payoffs increased (log odds of slope = −0.51, s.e. = 0.01, *p* < 0.001). Specifically, participants appeared indifferent between the two options when the sure payoff was roughly 2/3 of what they could have earned by choosing the uncertain option together (average indifference point = $9.98). Correspondingly, increasing SP values linearly decreased expected payoff as well (*p* < 0.001). Finally, risk attitudes as established from our lottery condition did explain some of this variance. Indeed, the more participants invested in the lotteries, the more likely they were to choose the uncertain option in the EG only (game^*^risk interaction: *p* < 0.001). However, the reported effects of similarity were net of the effect of all of these covariates.

## Discussion

Across across ages (Meltzoff, 2007), cultures (Apicella et al., 2012), and species (Massen and Koski, 2014) similarity has been shown to play a fundamental role in the formation of social ties and networks (McPherson et al., 2001; Ames, 2004; Mitchell et al., 2006; Fischer, 2009; Krueger et al., 2012; Fischer et al., 2013). Coordination problems have also perplexed decision theorists for decades (Schelling, 1960; Cooper et al., 1990; Van Huyck et al., 1990; Camerer, 2003; Heinemann et al., 2009). Yet no study has investigated whether classic coordination problems are affected by perceived interpersonal similarities (Cole and Teboul, 2004).

In this study we investigated this by having participants decide whether to take a number of real financial risks (as opposed to a safe alternative), when tacitly coordinating their choices with counterparts who were either similar or dissimilar to them with regards to three person-descriptive words. We report three novel findings: (1) in coordination games with strategic compliments, in which participants had an incentive to *match* their choices (stag hunts), similar counterparts incurred higher financial “risks” than dissimilar counterparts; (2) however, in games with strategic substitutes, where participants were to decouple their choices (entry games), we observed the opposite pattern: similar counterparts were willing to incur less risk than dissimilar ones; (3) both of these effects were only observed when similarity was based on traits that participants also liked and identified with. We would like to comment on each of these findings in turn.

### Similarity and “complementarity”: propensity for matching choices

Coordination games with strategic complementarities are not just a major theoretical problem for game theory (Camerer, 2003) they could also be a pragmatic one. The problem is that even in situations that present clear economic synergies to all players (i.e., typical “win-win” situations like stag hunts^22^), coordination still often fails (Cooper et al., 1990). Indeed, especially when the “risk” involved is high (Harsanyi and Selten, 1988)—that is, when the safe alternative to coordinating becomes large enough—coordination almost always fails (Cooper et al., 1992; Heinemann et al., 2009)^23^. Seemingly, this occurs because, even though all players would prefer to coordinate on the higher paying option^24^, they fear that their counterparts might not do the same^25^. Our results suggest that perceived interpersonal similarities could then provide the assurance^26^ players need in order to coordinate more efficiently: “I wish to choose the optimal option, and if my counterpart is like me he/she is more likely to do so as well.”

Notably, in our experimental design, similarity/dissimilarity was only based on self-reported identification with three trait-related words, and one could sensibly argue that this is no basis for a reliable estimate of similarity (or at least not enough to incur different financial risks for). In spite of this, even such a partial or occasional form of similarity proved sufficient to affect economic coordination. In fact, humans appear particularly sensitive to similarity-related cues, even to ones that are completely unrelated to the task at hand. For instance, notorious minimal-group paradigms have repeatedly shown how in-group favoritism and out-group discrimination in games can emerge even when the only thing that ingroups have in common is having preferred a painting of Kandinsky over Klee (Billig and Tajfel, 1973; or more recently, Chen and Li, 2009), rather than having over or under counted a number of dots on a screen (Gerard and Hoyt, 1974). In line with this, even non-social similarity (or “content free similarity”) has been shown to impact social inferences and perspective taking (Todd et al., 2010), as well as behavior in games (Mussweiler and Ockenfels, 2013)^27^. It thus appears that subjects can pick up on similarity-related cues rather easily and that they often then generalize them to unrelated domains^28^. Notably, a large meta-analysis on similarity (Montoya et al., 2008) suggests that, at least with respect to “relationship quality,” perceived similarities can even be more important than actual similarities, and this, in principle, could hold for coordination as well.

In line with this, we found that even our weak form of similarity not only decreased subjective uncertainty in stag hunts, but it also increased the expected payoff of the interactions. It follows that had the games been repeated, such similarity-related behaviors and cognitions could have been potentially been reinforced; and though this would be a matter for further experimentation, it opens the possibility that, regardless of whether generalizations of similarity are valid or invalid^29^, they could be adaptive in coordination environments with strategic complementarities.

### Similarity and substitutability: aversion to decoupling choices

Our hypothesis that similarity would provide assurance in stag hunt games was based on the following paraphrased inference: “I wish to choose the optimal option, and if my counterpart is like me he/she is more likely to do so as well.” However, and critically, while such a line of inference would indeed generate assurance in stag hunts, it could even increase uncertainty in games involving strategic substitutes, such as entry games. Indeed, in such games, if both participants choose their own optimal outcomes, they both obtain nothing at all. Our findings are in line with this: while similar players took more “risk” than dissimilar players in stag hunts, the opposite was true for entry games: similar counterparts took less risk than dissimilar ones.

Even though the direct comparison has seldom been made in the literature, previous findings suggest that games with strategic substitutes elicit higher uncertainty than games with strategic compliments (Chark and Chew, 2013). For instance, Camerer and Karjalainen (1994) found that players exhibited an aversion to uncertainty when they were to decouple their choices (i.e., because their payoffs were anti-correlated), while Fox and Weber (2002) observed a relative propensity for uncertainty when players had to match them (in a coordination game involving correlated payoffs). This could be related to the notion that stag hunts and entry games differ in amount of required deliberation and recursive thinking (Nagel et al., in preparation). In line with this, we find that, regardless of similarity, players clearly choose the uncertain option less frequently in entry games than stag hunts (see Figure 3,^30^) and that they choose it even less when they play entry games with similar counterparts.

A second important difference between stag hunts and entry games is that the standard notion of mixed strategy equilibrium (“MSE”) works very poorly for stag hunts but very well for entry games (Camerer and Fehr, 2006)^31^. For example, in an entry game with SP = 1, (risk-neutral) players are in MSE, only if 93% of them are “entering” (i.e., choosing the UP). Indeed, if a given player “*i*” believed that 93% of (non-*i*) agents were entering in such a game (or, equivalently, that a single counterpart “*j*” entered with a probability of 0.93), his *expected* earning for entering as well would be equal to the high payoff ($15.00) multiplied by the probability of being matched to someone who did *not* enter; that is, (1 - 0.93)^*^15.00 = $1.00. As one can see, for this specific probability only (*p* = 0.93), the expected value of entering is equivalent to the amount player *i* would obtain for sure by choosing the SP option. Consequently, for this expected entry rate (of other players), an agent would be indifferent between the two options, thus in equilibrium.

Naturally, this reasoning would seem futile because players have little information on which to base their probability estimates in one-shot entry games, so how can they decide who should enter and who choose the certain option? Yet, without communication, or trial and error, groups of players are known to “split up” in proportions that are very similar to those predicted by MSE (Camerer and Fehr, 2006; reviewed in Erev and Rapoport, 1998). Indeed, we observed this in our data as well: aggregating choices across participants, we found that MSE-based probabilities (computed for each SP^32^) predicted very well the proportions in which participants “split up” between entering and non-entering (*r* = 0.97, *p* < 0.001) (“To a psychologist” Daniel Kahneman said, “it looks like magic”) (Kahneman, 1988).

To this we make one addition: since MSE outcomes are actually rather inefficient in terms of payoffs^33^, and similarity lowered entry rates, similarity can actually increase the expected payoffs in entry games. In other words, since similarity generally lowered entry rates (in the ID+Like+ condition), the relatively fewer participants that *did* enter had a sufficiently high probability of being matched to someone who had not entered; high enough to significantly grant a payoff advantage of similarity. Notably, this occurred in spite of the fact that similarity did *not* increase participants' general ability to decouple their choices (while it did favor matching in the matching environment). We thus suspect that similarity might have “indirectly” increased expected payoffs in decoupling environments, more as a result of increased uncertainty (i.e., lowered entry rates), rather than as a result of increased decoupling abilities.

At any rate, our findings show how similarity can potentially increase expected payoffs of interactions over the two opposite poles of coordination: matching and decoupling. This could shed light on why homophily is so successful as a social attractor.

### The impact of similarity on coordination: not just social preferences

As illustrated by Cooper et al. (1990), “a weakness of the Nash equilibrium concept is that it may not generate a unique equilibrium. In this case it must be augmented by a hypothesis refining the *beliefs* of players about the strategies selected by their opponents” [cursive our own]. So far, we have treated perceived similarities as affecting precisely the beliefs or inferences of players in coordination environments: if similarity leads players to believe they are more likely to make similar choices, this should decrease uncertainty when choices are to be matched (stag hunts), but increase it when they are to be decoupled (entry games).

However, this interpretation faces a potentially important confound. Indeed, as we illustrated in the introduction of this paper, similarity has primarily been considered to increase interpersonal attraction (Byrne, 1971; McPherson et al., 2001) and a social preferences approach (Van Lange, 1999; Fehr and Fischbacher, 2003) could in principle explain our findings without recurring to inferences or beliefs at all. In fact, under the latter view, participants would simply “prefer” similar to dissimilar others and consequently choose the options that benefit similar targets more than dissimilar ones. In line with this, it can be demonstrated for our two games that if payoff interdependencies are introduced—such that one's subjective utility is assumed to be proportional to the payoff of one's counterpart (in addition to his/her own)—then the expected value of “risking” (relative to the alternative sure payoff) increases in SHs, but decreases in EGs, which is in fact the behavioral pattern we observe^34^.

However, our design enabled to address this potential confound. Specifically we found that similarity only affected coordination when the traits on which it was based on were also liked and identified with (our ID+Like+ condition). Importantly, had this effect been due to “liking” alone, we should have observed the reversed pattern of similarity in the ID+Like- or ID-Like+ conditions. In fact, in these conditions, dissimilar others were characterized by traits that the participants liked, whereas similar others were not (ID-Like+); or dissimilar others did not identify with traits that participants disliked, while similar counterparts did (ID+Like-). Had the effect of similarity in the ID+Like+ condition been due to liking alone, we should have thus found that risk rates were higher when participants coordinated with dissimilar others in stag hunts, and higher for similar others in entry games. However, neither of these effects was observed. This suggests that our observed effect of similarity on coordination was not due to liking alone.

## Conclusion

According to Quine (1969), “There is nothing more basic to thought and language than our sense of similarity”^35^ and psychologists have long insisted that, since early stages of development (Meltzoff, 2007), similarity could provide a fundamental window on to the minds of others (Ames, 2004; Robbins and Krueger, 2005; Goldman, 2006; Krueger et al., 2012). Indeed, the finding that similarity breeds attraction has been called “one of the most robust relationships in all of the behavioral sciences” (Berger, 1973). In spite of this, no study had investigated the impact of perceived similarities on tacit coordination.

Here, we demonstrated that agents are willing to incur higher financial risks when they are to coordinate their choices with similar others. However, we find that this effect is specific to coordination environments in which all agents would prefer to match their choices. In fact, when agents should decouple their choices, we observe the opposite effect; namely, similar others take less financial risks than dissimilar ones. On the basis of this, we suggest that perceived interpersonal similarities can indeed be used as a coordination device and that they can both decrease and increase strategic uncertainty, depending on the incentives at play. Furthermore, we find that when similarity is removed from interpersonal attraction, its impact on coordination is much decreased, if at all present. At last, our finding that perceived interpersonal similarities can increase the collected expected payoffs of agents might shed light on why homophily, or “love for similar others,” is so successful as a social attractor.

### Conflict of interest statement

The authors declare that the research was conducted in the absence of any commercial or financial relationships that could be construed as a potential conflict of interest.

## Footnotes

^1^Analogously, in conceited arguments, everyone would like to get their message through first, though if everyone tries to do so at the same time, no one succeeds, because attentional resources are limited. Or, in emergencies, everyone would tend to rush through the fire exit, though overcrowding it would only make things worse.

^2^Related measures have been proposed in terms of game “difficulty” (Rapoport, 1967), indexes of “correspondence” (Kelley and Thibaut, 1978), or game “harmony” (Zizzo and Tan, 2007).

^3^Camerer (2003) provides a number of real-life examples in which coordination is required but communication isn't possible. Furthermore, even when communication is possible, it doesn't completely resolve “matching problems” (Cooper et al., 1992) and it has even been shown to potentially complicate “decoupling” ones (Cooper et al., 1989; Ellingsen and Östling, 2010). Notably, if words have slightly different meanings to different people (i.e., different cultures), communication can also lead to incomprehension and conflict (Weber and Camerer, 2003).

^4^Though see Harsanyi and Selten (1988) or Carlsson and Van Damme (1993) for important game theoretic refinements on coordination.

^5^Eric Fischer's city maps are a particularly compelling demonstration of ethnicity-based homophily (https://www.flickr.com/photos/walkingsf/4981444199/in/set-72157624812674967) (though distinguishing homophilic aggregation from segregation might not be straightforward).

^6^One notorious instance of this is the “false-consensus effect” (see Marks and Miller, 1987 for a review); though see also Goldman (2006); Goldman (in particular Chapter 7) for a survey of all the (“high-level” mind-reading) domains in which subjects have been shown to attribute their own perspectives and knowledge to others.

^7^Intriguingly, humans have even been shown to recruit the same neural structures when answering questions about themselves and similar, but not dissimilar others (Mitchell et al., 2006; Jenkins et al., 2008).

^8^Notably, in notorious social dilemmas such as prisoner's dilemmas or public good games, cooperators aim to match their choices with their counterparts, while “free-riders” aim to decouple them. Indeed, many studies have stressed the strong link between cooperation and coordination (Dawes et al., 1977; Messé and Sivacek, 1979; Kollock, 1998a,b; Hayashi et al., 1999; Camerer, 2003). Consequently, if similarity has an effect on coordination, this could carry over to cooperation dilemmas (Fischer, 2009; Krueger et al., 2012; Fischer et al., 2013).

^9^Even newly hatched chicks have been shown to move toward a tone they had prenatally been exposed to rather than novel tones (Rajecki, 1974).

^10^Incidentally, in English, the same word can be used to express both concepts: “we like those who are like us.”

^11^More specifically, the choices of one's “average counterpart” were taken from the same experimental cell only for the similarity conditions. In the dissimilarity condition this did not seem justified. For instance, when coordinating with a dissimilar other in the Like+ID+ condition (or the Like+ID-, Like-ID+, and Like-ID- conditions), participants were matched with someone with who did not identify in the same triplet of traits, thus of someone in the Like+ID- (“dissimilar”) condition (and respectively, the Like+ID-, Like-ID-, and Like-ID+ conditions), not of someone in the Like+ID+ (“dissimilar”) condition. Correspondingly, averaged counterpart choices in the dissimilarity condition only were re-mapped as just described.

^12^In other words, the probability of successfully coordinating with others, when one chose the risky option, coincided with the “mean risk rate” of one's potential counterparts.

^13^ID+Like+: mean difference (ID - Liking) = 0.04, p > 0.3; ID-Like- (ID - Liking) = 0.07, 95% CI [− 0.007 0.1], p = 0.08.

^14^ID-Like+: mean difference (ID – Liking) = -3.4, p < 0.001; ID+Like- (ID – Liking): 3.7, p < 0.001.

^15^Mean difference in liking (ID-Like+) – (ID+Like-) = 3.7, p < 0.001; mean difference in liking (ID-Like+) – (ID-Like-) = 4.4, p < 0.001.

^16^Mean difference in liking (ID-Like+ - ID+Like+) = −1, p < 0.001.

^17^Log-likelihood difference_(*sim*−*dissim*)_ = 0.54, s.e. = 0.12.

^18^Log-likelihood difference_(*sim*−*dissim*)_ = −0.22, s.e. = 0.11.

^19^SH_(*sim*−*dissim*)_ = 0.05, s.e. = 0.11, *p* = 0.44; EG_(*sim*−*dissim*)_ = −0.13, s.e. = −0.11, *p* = 0.22.

^20^SH_(*sim*−*dissim*)_: −0.17, s.e. = 0.11, *p* = 0.13; EG_(*sim*−*dissim*)_: −0.01, s.e. = 0.11, *p* = 0.91.

^21^In fact, a model including all the conditions revealed a significant 3-way interaction between similarity, liking, and identity (*p* < 0.05), in determining coordination rates.

^22^Camerer (2003) calls the stag hunt game “the building block of economic situations with “strategic complementarities.”

^23^A potential parallel observation in prisoner's dilemma games is Rapoport's “game difficulty index” (Rapoport, 1967), which predicts higher cooperation depending on the ratio between payoff magnitudes of the “cooperate” or “defect” outcomes. This was a curious and early finding because, according to game theory, subjects should always defect in (one-shot) prisoner's dilemmas, thus “risk” should have nothing to do with it.

^24^In fact, when players can communicate their intentions to one another, they do coordinate on the higher paying option, even when they know that their “promises” are non-binding (Cooper et al., 1992).

^25^Furthermore, players might not only be afraid that their counterparts will not coordinate on the payoff efficient outcome (i.e., a 1st order belief), but they might also think that their counterparts fear the same about them (i.e., a 2nd order belief), and so forth (for higher order beliefs). This can generate what Hofstadter calls “reverberating doubt” (Hofstadter, 1985).

^26^Stag hunts are also called “assurance games” (Camerer, 2003).

^27^For example, in one study (Todd et al., 2010), participants were divided into two groups, both of which were shown a pair of images of landscapes. One group had to list the similarities between the two images while the other group had to list the dissimilarities. The two groups then underwent a number of social tasks involving other agents. One of the tasks, for instance, required participants to neglect their own (privileged) knowledge, in order to correctly infer the false-beliefs of a naïve target. Surprisingly, participants in the similarity condition had more difficulties in doing so, thus systematically (over-) attributing their own thoughts and perspectives to others.

^28^In spite of this, we don't expect similarity to always affect inferences, especially when it conflicts with other forms of relevant information. For instance, knowing that someone is very “risk-averse” would be arguably relevant to predicting his or her behavior in games involving some form of uncertainty. Consequently, we would expect similarity to no longer raise risk rates in stag hunts if it was, for instance, based on the fact that two players are similar in their “risk-aversion.” Similarities might also need to be salient enough, like group membership in related paradigms (Charness et al., 2006; Chen and Li, 2009).

^29^The case has often been made (Dawes, 1989; Krueger, 2008; Krueger et al., 2012) that some egocentric biases may actually not be biases at all, but the result of correct Bayesian inferences that are based on a sample size of 1 (i.e. one's own opinion, preferences or attitude).

^30^The two entry game curves are lower than the two stag hunt ones.

^31^MSE assumes that players choose options with a given probability, rather than choosing each strategy with dichotomous probability (i.e., *p* = 1 or *p* = 0)—as in “pure strategy” equilibrium. For instance, in the game of “rock, paper scissors,” there is only one mixed-strategy equilibrium, which is for both players to choose each strategy with a probability of 1/3 (Cook et al., 2012). In fact, if a player adopted any other probability distribution over his/her choices this would be exploited by a rational counterpart, thus leading the first player to revert back to a uniform distribution.

^32^We computed MSE solutions, for each SP value by solving for the “P_j_ (UP)” term in the following equation: SP = (1 - P_j_ (UP))^*^15.00; where P_j_ (UP) is the posterior probability of being matched with a counterpart who also chose the UP on the target trial.

^33^For instance, in a hypothetical EG with SP = 0 MSE would predict all “rational” agents to enter, “collide” with one another and thus to all earn 0. Consequently, many deviations from this inefficient equilibrium can raise average expected payoffs.

^34^Put differently, if one makes no inferences at all and simply wishes to choose the option that maximally benefits his/her counterpart, this option is the uncertain one in stag hunts, and the certain one in the entry game. Indeed, if one always made these respective choices in the two games, their counterparts would never lose.

^35^Though see Sloman and Rips (1998) or Gilboa and Schmeidler (1995) for more recent reviews on similarity.
