# Supplementary material for: The impact of perceived similarity on tacit coordination: propensity for matching and aversion to decoupling choices
Source: Front Behav Neurosci. 2015 Jul 28;9:202. doi: 10.3389/fnbeh.2015.00202 (PMC4516978; doi:10.3389/fnbeh.2015.00202)
Supplement: Supplementary file 1 [file Table1.PDF]

Supplementary Material

The impact of perceived interpersonal similarity on tacit coordination: propensity for matching and aversion to decoupling choices

Gabriele Chierchia[1,2]* and Giorgio Coricelli[2,3]

[1] Max Planck Institute for Human Cognitive and Brain Science, Leipzig, Germany; [2]Center for Mind/Brain Science, University of Trento, Italy; [3]Economics Department, University of Southern California, Los Angeles, USA

***Correspondence:**
Dr. Gabriele Chierchia
Max Planck Institute for Human
Cognitive and Brain Science
Department of Social Neuroscience
Stephanstraße 1A, 04103
Leipzig, Germany
chierchia@cbs.mpg.de

1. Supplementary Figures and Tables

1.1. Supplementary Table 1

To retrieve the participant-specific adjectives pertaining to each of the 4 groups of words (ID+Like+, ID+Like-, ID-Like+ and ID-Like-) (illustrated in section 2.2. of the study), an algorithm scanned the identity and liking scores of the 100 adjectives provided by each participant (scores were on a Likert scale from 1 to 7). For each group of words, the algorithm followed a different "search sequence". For instance, the values in the table below (Supplementary Table 1) represent the order in which adjectives of the ID+Like+ group were searched for. Since adjectives destined to the ID+Like+ group ideally had maximal identity scores (ID=7) and maximal liking scores (Like=7), the search sequence first looked whether there were any adjectives that satisfied this criterion. Correspondingly, in the table, there is a "1" at the intersection of the identity scores=7 and liking scores=7 (bottom right corner of the table).

The algorithm was set to stop once it had collected 3 words that satisfied the score criterion mentioned above. However, it could occur that participants had not provided this exact combination of scores (i.e. ID=7, Like=7) for 3 of the 100 adjectives. If this occurred, the algorithm stored any words that had satisfied the criterion and then searched for the "next best" adjective. For the ID+Like+ group, such a "next best" adjective is one for which the participant had provided an ID score of 7 and a liking score of 6. Correspondingly, in the table, this combination of ratings (ID=7, Like=6) is 2nd in the search sequence. The algorithm then kept searching for adjectives in the order established by supplementary table 1, until it retrieved 3 adjectives. These 3 adjectives were the ones that maximized the participant's identity and liking scores, and that ultimately composed this participant's ID+Like+ group.

To obtain the search sequence of the other groups of words it is sufficient to rotate the values in the matrix below: a 90° clockwise rotation of the ID+Like+ search sequence values (in Table 1 below) provides the corresponding values of a search sequence for words destined to the ID+Like- condition, a counterclockwise rotation provides the ones for the ID-Like+ group, and a 180° rotation provides the ones for the ID-Like- group.

Once the four triplet of traits was identified for each participant (ID+Like+, ID-Like+, ID-Like+, ID-Like-), the (four) corresponding identity ratings of alleged counterparts were generated as follows: in the similarity condition, for each triplet and for each participant, the algorithm randomly selected 2 of the 3 identity scores and randomly jittered the remaining score by 1 (in any of the available direction, i.e. higher or lower); in dissimilarity conditions, 2 identity scores of one's counterparts were placed on the maximum (or minimum) *opposite* side of the identification rating bar and the remaining (randomly designated) score of the 3 was increased (or decreased) by 1. For instance, if a triplet of traits had been selected for participant "i" (i.e. "organized, funny and talkative") and *i* had provided the following identity ratings to each item in the triplet: (6, 6 and 7); then the identity scores of i's counterpart (for the same three words) could have been 6, 7, 7, in the similarity condition and 1, 2, 1 in the dissimilarity condition.

Liking Scores

Identity Scores: ID+Like+	1	2	3	4	5	6	7
1	49	48	46	44	42	40	38
2	47	36	35	33	31	29	27
3	45	34	25	24	22	20	18
4	43	32	23	16	15	13	11
5	41	30	21	14	9	8	6
6	39	28	19	12	7	4	3
7	37	26	17	10	5	2	1

Supplementary Table 1. The adopted search sequence for adjectives destined to the Like+ID+ group. The algorithm first searched for any adjectives that a target participant had provided maximum ID scores and maximum liking scores to (correspondingly number "1" appears at the bottom right corner of the matrix, that is for maximum ID ratings -7- and maximum Liking ratings -7). If the algorithm already found 3 adjectives in this first cell, it stopped. Otherwise, it stored any adjectives it had found and then searched for the "next best" adjective, namely one for which the subject had provided an ID score of 7 and a Liking score of 6. Correspondingly, "2" appears at the intersection of these identity and liking scores (i.e. 2 = ID, Like (7,6)). The algorithm proceeded in this manner, until 3 adjectives had been collected, then it stopped.

1.2. Supplementary Table 2

Analysis of deviance table for a generalized logistic mixed model described in section 2.6.2 of the study.

GLMM	Chi-square	Df	Pr(>Chi-square)
sure payoff	3183.695	1	0***
game	853.223	1	0***
similarity	1.222	1	0.269
liking	0.415	1	0.519
identity	1.141	1	0.285
risk (lottery)	0.461	1	0.497
sure payoff*game	79.901	1	0***
game*similarity	4.857	1	0.028*
similarity*liking	0.007	1	0.936
game*liking	0.239	1	0.625
similarity*identity	0.194	1	0.66
game*identity	1.691	1	0.194
liking*identity	0.035	1	0.852
game*risk (lottery)	396.706	1	0***
game*similarity*liking	12.665	1	0***
game*similarity*identity	2.263	1	0.133
similarity*identity*liking	8.873	1	0.003**
game*liking*identity	1.41	1	0.235
game*similarity*liking*identity	5.046	1	0.025*

Supplementary table 2. Analysis of Deviance Table (Type II Wald chi-square tests) for a generalized logistic mixed model (GLMM) estimating the probability risky choices. *=p<0.05, **=p<0.01, ***=p<0.001.

1.3. Supplementary Table 3

	MATCHING (stag hunt)		DECOUPLING (entry game)	
	Similarity	Dissimilarity	Similarity	Dissimilarity
ID+Like+	0.63 (0.03)	0.56 (0.03)	0.38 (0.03)	0.4 (0.03)
ID+Like-	0.58 (0.04)	0.6 (0.03)	0.4 (0.03)	0.4 (0.03)
ID-Like+	0.59 (0.03)	0.59 (0.03)	0.38 (0.03)	0.39 (0.03)
ID-Like-	0.61 (0.03)	0.58 (0.03)	0.4 (0.03)	0.35 (0.03)

Supplementary table 3. Averaged "risk" rates in each of our experimental cells. We first aggregated risk rates across sure payoffs for each participant separately. Then we aggregated these means over the different participants. In parenthesis is the standard error of the latter means. The left-most column indicates whether participants strongly identified with the adjectives that the similarity was based on (ID+) or did not identify with them (ID+), as well as whether the adjectives were liked (Like+) or disliked(Like-).

2. Instructions

Participants had instruction sheets available from the beginning of the experimental session until the end. However, a blank page separated each section of the instructions, with large bold characters saying, "Please do not read past this point until you are instructed to". This was also announced orally. The reason for this was that we did not want participants to know about the games before rating the personality traits. Instructions were read out loud by the experimenters, one section at the time, and participants could follow on their own sheets.

In what follows, we provide the instructions we used:

Welcome,

And thank you for participating in this experiment on decision-making.

You will be paid for your participation in cash at the end of the experiment. The entire experiment will take place through computer terminals, and all the interactions between participants will take place through the terminals. You will remain anonymous to all the other participants during the entire experiment and afterwards.

Just for participating you will earn a show up fee of $5.00 dollars. Any further payment will depend on the decisions you and others make throughout the session. You will be able to earn up to $30.00 dollars. We remind you that your participation is completely voluntary and you can leave the session at anytime. In this case however we will be unable to re-admit you to the session and you will receive the show up fee only.

The session will consist of 3 sections. Specific instructions will be handed out prior to each. These are the names and preliminary descriptions of the sections:

1) "Traits" (20 min)
In which you will evaluate a number of personality traits/characteristics.

2) "Interactions" (35 min)
In which you will interact with anonymous counterparts in two interaction variants.

3) "Urn" decision (5 min)
In which you will decide whether and how much to invest on a random extraction.

We will read all instructions out loud. If you have any questions raise your hand and your question will be answered so that everyone can hear. If any difficulties arise during the experiment, raise your hand, and an experimenter will come and assist you.

Section 1: "Traits"
The first task consists of a questionnaire. On each page you will a list of personality-related traits, such as "organized", "impulsive" etc. At the top of the page a question will inform you on how to evaluate each one. There will be 2 questions, so always make sure to check what the question at the top of the screen asks before responding. You will respond by giving scores on a rating bar. Scores go from 1 to 7, where 1 means "absolutely not", 7 means "extremely", and 4 indicates indifference. It is very important for us that you respond as honestly and naturally as possible. We remind you that your answers will remain anonymous and we will have no way of tracking them back to you. Any questions?

Section 2: Interactions

For the following interactions you and a matched counterpart will be required to make a choice between the *same 2 options,* A and B, respectively on the left and the right side of the screen (see fig. 1 and 2).

You will see several screens. In each one there will be several different A options and one B option. For each screen you will be matched with one anonymous counterpart in the same room, and he/she will be matched with you.

You will take part in 2 different types of interactions, identified only by the words in the B option, on the right side of the screen. It is very important that you carefully consider the words in the B option to understand what type of interaction you're in. The two interactions are represented as so: "$15.00 **IF BOTH** choose B" represents one type of interaction; and "$15.00 **IF ONLY YOU** choose B" represents the other.

In both interaction types you will have to choose between A and B several times with one counterpart. If you choose A, you will receive A no matter what your counterpart chooses. If you choose B, the outcome will depend on your counterpart's decision and on the type of interaction:

In "$15.00 **IF BOTH**", **both you and your counterpart will receive $15.00 only if you BOTH choose option B. If you choose option B and your counterpart chooses A you will receive 0** and he/she will still receive A. Vice versa, if you choose A and your counterpart chooses B, you will receive A and your counterpart will receive $0.00.

In "$15.00 **IF ONLY YOU**", you will receive $15.00 **only if you choose B and your counterpart chooses A. If both you and your counterpart choose B you will both receive $0.00.** If you choose A and your counterpart chooses B, your counterpart will receive $15.00 and you will receive A.

We remind you that you and your counterpart will have the exact same rules in both interaction types, and you will view the exact same options A and B.

You will not be told the outcome of any of your decisions until the end of the experiment. At the end of the experiment a program will extract one trial at random. You will be paid on the basis of this trial. For this trial you will also be told: which decision it was, how you responded, and how your counterpart responded. However, since you won't know which decision will be paid until the end of the experiment we strongly suggest that you pay attention to each decision you make.

You will now take a brief quiz to see if everyone has understood these instructions correctly. This is also done so that everyone can be sure that everyone else understood the task correctly.

Are there any questions?

Payment for A:	Choose A or B:	Payment for B:
$14.00	A ◯ ◯ B	$15.00 IF BOTH choose B<br>$0.00 IF ONLY YOU choose B
$3.00	A ◯ ◯ B	
$11.00	A ◯ ◯ B	
$10.00	A ◯ ◯ B	
$4.00	A ◯ ◯ B	
$9.00	A ◯ ◯ B	
$2.00	A ◯ ◯ B	
$1.00	A ◯ ◯ B	
$13.00	A ◯ ◯ B	
$12.00	A ◯ ◯ B	
$15.00	A ◯ ◯ B	
$5.00	A ◯ ◯ B	
$6.00	A ◯ ◯ B	
$8.00	A ◯ ◯ B	
$7.00	A ◯ ◯ B	

FIG. 1.

Payment for A:	Choose A or B:	Payment for B:
$14.00	A ◯ ◯ B	$15.00 IF ONLY YOU choose B<br>$0.00 IF BOTH choose B
$3.00	A ◯ ◯ B	
$11.00	A ◯ ◯ B	
$10.00	A ◯ ◯ B	
$4.00	A ◯ ◯ B	
$9.00	A ◯ ◯ B	
$2.00	A ◯ ◯ B	
$1.00	A ◯ ◯ B	
$13.00	A ◯ ◯ B	
$12.00	A ◯ ◯ B	
$15.00	A ◯ ◯ B	
$5.00	A ◯ ◯ B	
$6.00	A ◯ ◯ B	
$8.00	A ◯ ◯ B	
$7.00	A ◯ ◯ B	

FIG. 2.

Interactions with traits

[The section below was read after participants took part in the questionnaire probing their understanding of the game rules and after the “trait-neutral” games].

Now you will take part in the interactions previously explained. The only difference is that, this time, you will possess information about your counterpart, and he/she will possess information about you.

This occurs as follows: before interacting with each counterpart you will see a screen with 3 characteristics (FIG. 3), and the words “YOU” and “OTHER”. You will be shown how much your counterpart is represented by these characteristics, by looking at the rating bars under the word “OTHER”. The rating bars under the word “YOU” are a reminder, they are the responses *you* gave to the same characteristics. Your counterpart will be seeing how much you are represented by the exact same characteristics.

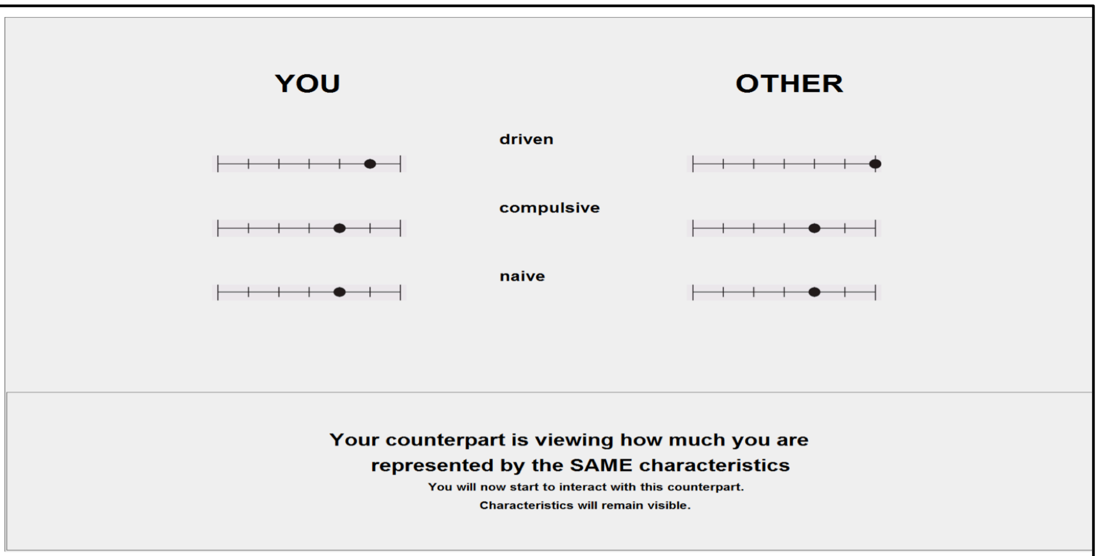

FIG. 3

Examine the available characteristics about your counterpart and you. This screen will disappear automatically. In the next screen you will be interacting with this counterpart (FIG. 4) in one of the two types of interactions (if BOTH or if ONLY YOU), the characteristics will remain visible.

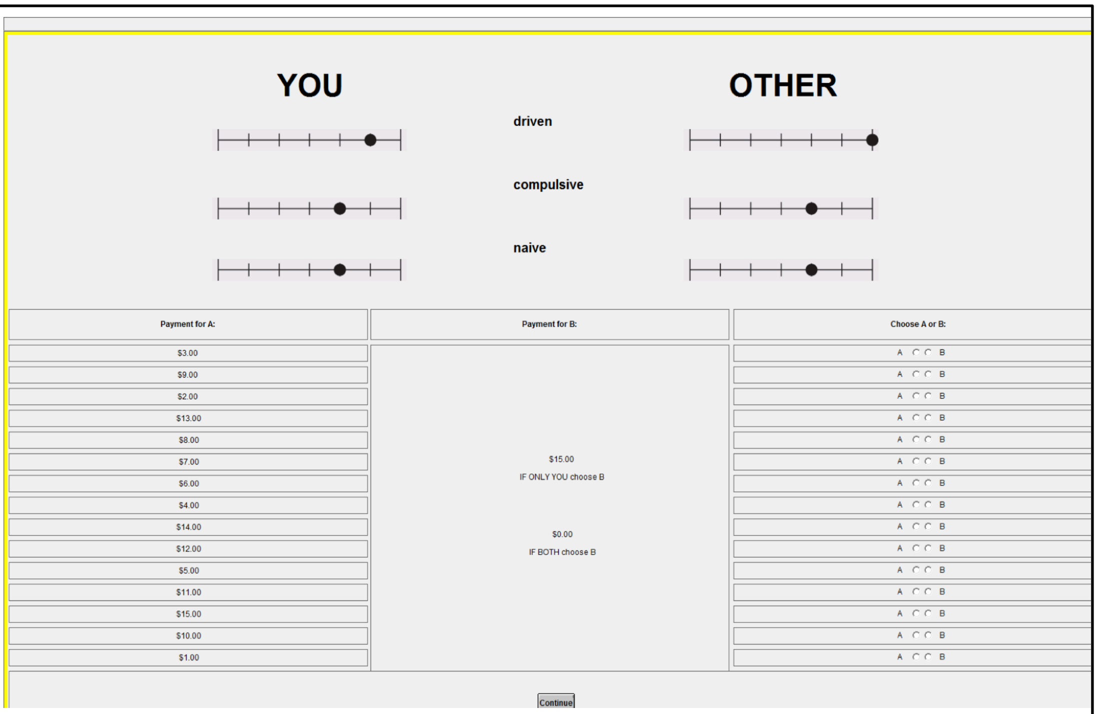

FIG. 4. A screenshot of the main task of our study (see section 2.1 and 2.2. of the paper).

"URN" decision

We have prepared a physical urn, as all you have seen. In it, there are 2 "RED" and 1 "BLUE" object. At the end of the session one extraction will be made.

In the following decision you will be asked: "How much do you bet on RED?"

For this decision you will receive $5.00. You can choose any amount X, with X between 0 and 5 dollars, 0 and five included. If you choose 0, you will receive $5.00 dollars, no matter which color is extracted. If you invest any non-zero amount X, you will receive the double of X only if RED is extracted, and you will lose X otherwise.

For instance if you choose 5 dollars, and RED is extracted you will earn $10.00; if however BLUE is extracted you will lose the 5 dollars.

If you invest $1.00 and RED is extracted you will obtain $2.00. If BLUE is extracted you will lose $1.00 dollar. This means that at the end of the task your earnings will be the following:

$6.00 (if RED is extracted) = $5.00 (initial endowment) - $1.00 (choice) + $2.00 (win);

or:

$4.00 (if BLUE is extracted) = $5.00 (initial endowment) - $1.00 (choice).

Are there any questions?

Earnings screen

After making the “URN” decision we will make the extraction from the urn. One of you will type the extracted value into his/her terminal.

The following screen will show the outcome and earnings for: the extracted interaction decision and the urn decision. A final box will show your total earnings for the experiment.

Once you have observed your earnings you can press the button at the bottom right of the screen. This will make your earnings disappear from the screen so that they will remain private. The Lab Manager will then call you by name for you to come and pick up the corresponding payment.

Thank you very much for your participation.
